# Insights Into Electronic-Cigarette Perspectives: Smokers’ Knowledge Attitudes and Practices at Hamad Medical Corporation, 2020

**DOI:** 10.2147/SAR.S473116

**Published:** 2025-08-30

**Authors:** Mohamad Chehab, Yasamin Abdu, Noora J Alkubaisi, Ahmad Al Mulla, Mohamed Iheb Bougmiza

**Affiliations:** 1 Community Medicine Department, Hamad Medical Corporation (HMC), Doha, Qatar; 2 Psychiatry Department, Sheffield Health and Social Care, NHS Foundation Trust, Sheffield, United Kingdom; 3 Community Medicine Department, Primary Health Care Corporation (PHCC), Doha, Qatar; 4 Department of Medicine, Hamad Medical Corporation, Doha, Qatar; 5 Community Medicine Department, College of Medicine, Sousse University, Sousse, Tunisia; 6 Community Medicine Department, College of Medicine, Qatar University, Doha, Qatar

**Keywords:** electronic cigarette, smoking cessation, smoking, tobacco, knowledge

## Abstract

**Purpose:**

The rising use of electronic cigarettes alongside traditional tobacco presents a global health concern. Despite this, little is known about smokers’ knowledge and attitudes toward electronic cigarettes in Qatar. This study addresses this gap by exploring the knowledge, attitude, and practice (KAP) of electronic cigarette use among smokers attending cessation clinics in Qatar.

**Patients and Methods:**

This analytical cross-sectional study involved 453 adult smokers, recruited through systematic random sampling at the Tobacco Control Center (TCC) in Qatar in 2020. Participants were interviewed via phone using a structured 42-item questionnaire after providing verbal consent. A cut-off score of 3 or higher indicated good knowledge, while a score of 1 or higher reflected a positive attitude. Bivariate analyses followed by logistic regression identified predictors of good knowledge and electronic cigarette use.

**Results:**

Of the 453 participants, the average age was 38.9 ± 8.9 years, with most being male (95.1%, n=429), married (77.9%, n=353), and university-educated (64.7%, n=293). Participants had smoked for an average of 18.5± 9.1 years, consuming 15.3 cigarettes per day. Low nicotine dependence was found in 36% (n=163), while 5.5% (n=25) were highly dependent. About 60.2% (n=259) demonstrated good knowledge of electronic cigarettes, but 69% held negative attitudes ((n=289). Nearly half (48.3%, n=219) had tried electronic cigarettes, with 55.2% using them for cessation attempts (n=121). Age, marital status, education, and income levels were significantly associated with knowledge and practice, with high income (≥ 30,000 QR/month) predicting both good knowledge and use.

**Conclusion:**

Although smokers are generally aware of electronic cigarettes, gaps persist in understanding their contents and health effects. Negative attitudes persist, yet half of current smokers have experimented with them. Tailored education and regulations are needed to dispel misconceptions and minimize risks for smokers in Qatar.

## Introduction

Tobacco consumption accounts for 7 million global deaths annually due to direct use (6 million) and (1.2 million) due to passive smoking.^1^ Furthermore, the economic burden of tobacco use has overwhelmed nations and healthcare systems. Similarly, direct and indirect costs of tobacco usage have totaled 1.852 trillion dollars or 1.8% of the global gross domestic product.^2^ In Qatar, the most common forms of tobacco are cigarettes (42.8%), waterpipes (20.9%), and medwakh (3.2%), according to a 2019 study of governmental employees and university students. Alternative tobacco products like smokeless tobacco (1.9%), electronic cigarettes (2.0%), and heat-not-burn products (0.3%) were less frequently used.^3^ Moreover, several effective tobacco control measures were adopted by Qatar, including tobacco taxes, the designation of smoke-free areas, anti-smoking health awareness, and the expansion of free or subsidised tobacco-dependence treatment services, such as smoking cessation clinics at Primary Health Care centres and Hamad Medical Corporation Tobacco Control Center.^3^ These clinics offer tailored support through behavioural counselling and pharmacotherapy, including Varenicline and Nicotine Replacement Therapy (NRT). A study conducted among 759 smokers who attended these clinics between January 2019 and June 2020 revealed a 30-day quit rate of 32.4% at six months follow-up.^4^

Electronic nicotine delivery systems (ENDS) and electronic non-nicotine delivery systems (ENNDS) heat liquids to produce aerosols for inhalation. ENDS contain nicotine, while ENNDS are marketed as nicotine-free, though this may not always be accurate. These systems come in various forms, such as e-cigars, e-pipes, and e-hookahs, with e-cigarettes being the most widely used type.^5^ ENDS is an electronic system that delivers nicotine by aerosol upon heating a nicotine-containing solution.^6^ The product is delivered through various devices (disposable, rechargeable, modular), flavours, and nicotine content typically ranging between 3 and 36 mg/mL.^7^

The use of electronic cigarettes has increased during the last decade. A review of surveys from 2019 about the prevalence of electronic cigarette use among the general adult and young populations in Europe, found that the prevalence of current electronic cigarette use ranged from 0.2% to 27%, ever-use ranged from 5.5% to 56.6%, and daily use ranged from 1% to 2.9%.^8^

Advocates of electronic cigarettes claim that the absence of any combustion process makes them safer than classical cigarettes. Subsequently, it has been suggested that it is a candidate tool for smoking cessation assistance among current smokers.^9–12^ On the other hand, evidence in the literature remains questionable as to whether electronic cigarettes can serve the means mentioned above or not. Moreover, the health effects of such devices on consumers and the public remain understudied.^13^ For example, it has been reported that using electronic cigarettes hurts wound healing among surgical patients.^14^ However, the evidence was limited in any associated long-term effects or carcinogenic risks. Additionally, the passive exposure to vapour from electronic cigarettes, known as Second-hand aerosol, is also not benign.^15^ A systematic review found that second-hand aerosol contains elevated nicotine levels and other compounds, such as formaldehyde and metals.^16^

Reviewing the literature consistently shows a positive association between electronic cigarette use and an increased risk of cigarette smoking onset.^17–19^ A potential explanation for this link is the “common liability” theory, which posits that the association is due to a shared predisposition for using tobacco products rather than a direct causal relationship.^20^,^21^ This predisposition, influenced by genetic and behavioural factors, may lead individuals to use various substances, including tobacco and psychoactive products. Supporting evidence shows that the use of other tobacco products (eg, cigars, hookahs) or substances like alcohol and cannabis also predicts cigarette smoking onset, with the risk increasing as more types of tobacco products are used.^19^,^20^,^22^,^23^

On the other hand, the World Health Organization (WHO) have flagged several concerning factors, such as the safety of these devices, their impact on nicotine dependence, and their relation to future tobacco use.^24^ Moreover, in concurrence with this increasing use of electronic cigarettes among smokers of different age groups, ninety-eight nations have developed relevant national regulations on the sale, advertisement, packaging, product regulation, taxation, and surveillance.^25^ Qatar signed and ratified the WHO Framework Convention on Tobacco Control (FCTC) in 2004, and to date, the ongoing implementation of the treaty is high. The updated law no.10 in 2016 on tobacco control prohibits the country’s manufacturing, importing, selling, displaying and distributing electronic cigarettes.^26^,^27^ However, despite these efforts, a recent study that investigated the use, knowledge, and attitudes toward electronic cigarette usage among National University students in Qatar found the prevalence of electronic cigarette use to be 14%.^28^

Data about smokers’ knowledge, attitudes, and practices regarding electronic cigarettes and their associated factors is limited in Qatar. The current research aimed to Determine the knowledge, attitude, and practice regarding electronic cigarettes among smokers attending the smoking cessation services in Hamad Medical Corporation. This will help identify any existing gap among that population and guide future intervention strategies.

## Materials and Methods

### Study Design, Setting and Population

We conducted a telephone-based analytical cross-sectional study in the smoking cessation clinics under the Tobacco Control Centre (TCC), a WHO collaborating centre for treating tobacco dependence at Hamad Medical Corporation. The centre treats approximately 1200 new patients annually, each attending around six appointments.^29^ The health services these clinics provide are either subsidised or free to the entire registered population, both nationals and expatriates. Therefore, the target population included current smokers aged 18 and above, either Arabic or English speakers, who attended those clinics by phone during the study period from September to December 2020. In this study, no exclusion criteria were applied. All participants who met the initial inclusion criteria were enrolled.

### Study Procedure

After obtaining the study approval from the Institutional Review Board (IRB) at Hamad Medical Corporation (MRC-01-19-464), and since all the smoking cessation clinics were included in the current study, a systematic random sampling technique of smokers was employed to enrol the required number of eligible participants. Thus, a list of the smokers attending each of these clinics was obtained monthly from the TCC-HMC administration and was sampled accordingly, where every third smoker was contacted via telephone to participate in the study until the sample size was fulfilled. After explaining the aim and purpose of the study, verbal consent was elicited, and if consenting, the participant underwent the telephonic interview-based questionnaire for an average of 5–10 minutes.

The sample size was calculated using the following formula:^30^ N = Z ^2^ P (1 - P)/ d ^2^ where N represents the sample size, Z is the confidence level statistic (1.96 for the 95% confidence interval), d is the precision (0.05), and P is the expected prevalence (we used the prevalence of knowledge about electronic cigarettes of 49%, which was obtained from Qatar’s earlier Global Adult Tobacco Survey 2013).^31^ Thus, the calculated sample size was 384. Considering an expected non-response rate of 20%, the estimated sample size based on this calculation was 460.

### Study Instrument

The researcher developed a structured questionnaire, and its face validity was established through an extensive literature review and consultation with experts from the TCC. We used a questionnaire comprised of forty-two items, including seven on socio-demographic factors, nine on the participants’ smoking history, and twenty-six on smokers’ knowledge, attitude, and practice (KAP) towards electronic cigarettes. The KAP items were divided into eight items, ten items, and eight items among the knowledge, attitude, and practice components. The format of the questions varied between multiple choice, dichotomous (yes/no), closed, and Likert scale questions. The questionnaire was prepared in English and translated into Arabic with a bilingual translator’s back translation. Before data collection, a pilot study was conducted among a convenient sample of 20 participants from one TCC clinic; the piloted 20 participants were omitted from the study database.

### Outcome Measures

#### Variables Regarding the Knowledge of Electronic Cigarettes

The participants’ knowledge of electronic cigarettes was evaluated through eight questions, six of which (except questions 3.b., 3.c.) were scored. Each correct answer was rewarded one point, and the total continuous knowledge score was 6. Thus, the participants were categorised as having “poor knowledge” if their total score was ˂3 and “good knowledge” if the total score was ≥ 3.

#### Variables Regarding the Attitude Towards Electronic Cigarettes

The respondents’ attitude towards electronic cigarettes was assessed through ten questions, most of which included a four-point Likert scale answer between “strongly agree”, “agree”, “disagree”, and “strongly disagree”. Overall, the attitude score was calculated through individual points granted to each question with a total attitude continuous score of +17 and a minimum of −17. Based on the median split method, those with scores below 0 were categorised as having a negative attitude, while those with scores 0 and above were categorised as having a positive attitude.^32^

#### Variables Regarding the Practice of Electronic Cigarettes

Regarding the practice of participants, it was assessed through multiple indicators,^33^,^34^ such as ever-user of electronic cigarettes (tried or used an electronic cigarette in their life), current user of electronic cigarettes (having used an electronic cigarette in the past 30 days), and daily user of electronic cigarettes (reporting daily use of electronic cigarettes).

### Data Analysis

Descriptive statistics regarding mean and standard deviation (SD) for quantitative variables and frequencies and percentages were calculated for categorical variables whenever appropriate. The Chi-square test (X2) was used to assess the association between the independent variables and the outcome measures with the Monte Carlo method to accommodate independent variables with more than two categories. Finally, variables who showed significance in the bivariate analysis (p value≤ 0.05) were entered in the multivariate logistic regression models to calculate the adjusted odds ratio (aOR) with their corresponding 95% confidence interval (CI), in order to assess the predictors for the outcome. However, the analysis was not pre-registered, and the results should be considered exploratory.

## Results

During the data collection period (September to December 2020), we approached 1188 adult current smokers to participate in the study. Of these, 314 individuals declined to participate, 421 did not respond to our calls, and 453 agreed to participate.

### Socio-Demographic and Smoking-Related Characteristics

Participants’ mean age (± SD) was 38.9 ± 8.9 years. Most participants (95.1%, n=429) were men, with 77.9% being married (n=353). Furthermore, 64.7% held a university degree (n=293), and 87% were employed (n=394). Income levels ranged from less than 10,000 to 30,000 Qatari Riyals per month. The mean duration (± SD) of smoking was 18.5 ± 9.1 years. Among those who smoked daily (92.5%, n=419), the mean number (± SD) of cigarettes per day was 15.3 ± 10.8.

Regarding nicotine dependence based on the Fagerström Test for Nicotine Dependence (FTND), 36% (n=163) had low dependence (scores 1–2),^35^ while 5.5% (n=25) were highly dependent (scores ≥ 8).^35^ Concerning FTND responses, 48.3% (n=219) reported smoking ten or fewer cigarettes daily (FTND −1). For the duration before smoking the first cigarette of the day (FTND −2), 36.0% (n=163) reported waiting more than sixty minutes after waking up. When asked about the most challenging cigarette to quit (FTND −3), participants were almost equally split between the waking up cigarette (50.1%, n= 227) and any other cigarette (49.9%, n=226). Additionally, (62.7%, n=284) denied smoking more frequently during the first hours of the day than the rest of the day (FTND −4), while (59.6%, n=270) reported stopping smoking when very ill (FTND −5). Furthermore, (69.3%, n=314) denied having difficulty refraining from smoking in prohibited areas (FTND −6), as shown in Table 1.

**Table 1:** Smoking Characteristics of Adult Current Smokers at the Tobacco Control Center-Hamad Medical Corporation in Qatar During 2020 (n=453)

Smoking Characteristic	n (%)
Earlier quit attempt(s)	426(94.0)
Daily tobacco smoker	419(92.5)
Type of tobacco used:	
Regular cigarettes	453(100.0)
Regular shisha	20(4.4)
Sweika	4(0.4)
Midwakh	2(0.9)
Number of cigarettes per day (FTND −1):	
Ten or less	219(48.3)
11-20	175(38.6)
21-30	30(6.6)
31 or more	29(6.4)
Time before smoking a first cigarette (FTND −2):	
Within 5 minutes	106(23.4)
6 to 30 minutes	124(27.4)
31 to 60 minutes	60(13.2%)
More than 60 minutes	163(36.0)
Most difficult cigarette to quit (FTND-3):	
Morning (waking up) time cigarette	227(50.1)
Any other	226(40.9)
Smoking more frequently during the first hours of the day (FTND-4)	169(37.3)
Smoking when very ill (FTND-5)	183(40.4)
Difficulty to stop smoking in forbidden places (FTND-6)	139(30.7)
Ever used an electronic cigarette	219(48.3)
Use of electronic cigarettes for smoking cessation	121(55.2)
Use of electronic cigarettes in the past 30 days	7(3.2)
Daily electronic cigarette user	1(14.3)
Surrounded or live with e-cigarette users	108(23.8)

**Abbreviation**: FTND, Fagerström Test for Nicotine Dependence.

### Participants’ Knowledge of Electronic Cigarettes

The participants exhibited a mean total knowledge score (±SD) of 3.13 (±1.7) regarding electronic cigarettes. The majority (60.2%, n=259) demonstrated good knowledge, scoring three or higher. Nearly all participants (94.9%, n=430) were aware of electronic cigarettes, yet (59.6%, n=255) rated their knowledge as minimal. Interestingly, (72.5%, n=307) relied on family members or friends for information on electronic cigarettes.

Regarding specific knowledge items, 51.2% knew electronic cigarettes were available in multiple forms (n=220). However, a significant proportion remained uncertain whether electronic cigarettes contained tobacco (58.6%, n=252) or carcinogens (52.8%, n=227). Furthermore, 59.5% (n=256) of participants needed to be aware of the second-hand aerosol concept. Table 2 presents a detailed breakdown of these findings.

**Table 2:** The Knowledge of Electronic Cigarettes Among Current Smokers at the Tobacco Control Center-Hamad Medical Corporation in Qatar During 2020 (n=453)

Knowledge Component	n (%)
Heard about electronic cigarettes	430(94.9)
Self-rating of knowledge level	
Minimal	255(59.6)
Moderate	128(29.9)
Extensive	45(10.5)
Source of knowledge about electronic cigarettes	
Family or friends	309(72.5)
Facebook/ YouTube/ other social media networks	69(16.2)
Internet (websites or blogs)	38(8.9)
Journals and newspapers	5(1.2)
TV or radio	5(1.2)
Electronic cigarettes come in more than one form:	
Yes	220(51.2)
No	17(4.0)
I do not know	193(44.9)
Tobacco, a constituent of electronic cigarettes:	
Yes	52(12.1)
No	126(29.3)
I do not know	252(58.6)
Presence of nicotine in electronic cigarettes	
Yes	202(47)
No	20(4.7)
May or may not	23(5.3)
I do not know	185(43.0)
Presence of carcinogens in electronic cigarettes	
Yes	186(43.3)
No	17(4.0)
I do not know	227(52.8)
Contents of electronic cigarettes’ vapor:	
Only water vapor	14(3.3)
Water vapor and other chemicals	160(37.2)
I do not know about Second-hand aerosol	256(59.5)

### Participants’ Attitudes Towards Electronic Cigarettes

**Figure 1:**
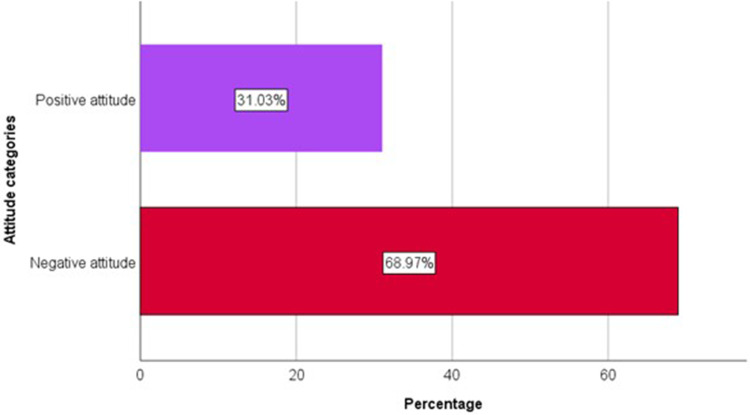
Distribution of smokers by attitude towards e-cigarettes (n=419).

The participants’ mean attitude score was −2.9 (±4.9), indicating a prevailing negative sentiment towards electronic cigarettes among more than two-thirds (69.0%, n=289) of smokers, with scores falling below zero, as seen in Figure 1. Nearly half of the respondents (48.1%, n=219) reported having experimented with electronic cigarettes.

Opinions regarding electronic cigarettes were divided: 52.8% agreed or strongly agreed that they were addictive (n=235), while 52.2% believed they could assist in smoking cessation (n=236). However, a significant majority (84.5%, n=381) perceived second-hand vapour as detrimental to human health, contrasting with 36.4% who considered electronic cigarettes less harmful than traditional cigarettes (n=165). Regarding social perceptions, (55.8%, n=252) of participants agreed or strongly agreed that electronic cigarettes enhanced social acceptance. Opinions on whether electronic cigarettes provided pleasure were evenly split, with 49% in support (n=213) and 51% opposed (n=222). Regarding usage regulations, 65.1% of respondents were against using electronic cigarettes in smoke-free areas (n=295), while 83.6% supported electronic cigarette regulation (n=377). Additionally, 86.3% of participants opposed the promotion of electronic cigarettes (n=390).

### Participant’s Use of Electronic Cigarettes

Almost half (48.3%, n=219) of the people in the study said they had tried using electronic cigarettes before. Among those who had tried them, more than half (55.2%, n=121) used them to try to quit smoking. However, only a few (3.2%, seven people) had used them in the past month.

Out of those seven current users, all of them considered themselves electronic cigarette users, and on average, they had been using them for about 18 months. Two of them knew how much nicotine they were using (between 1 to 11 milligrams), but the others were not sure. A few of the current users (14.3%, which is one person) said they used electronic cigarettes every day. Additionally, about a quarter (23.8%, which is 108 people) of the smokers in the study said they were often around people who used electronic cigarettes.

Among smokers who used electronic cigarettes to try to quit, about a quarter (23.1%, which is 28 people) were successful. Still, nearly half (46.3%, which is 56 people) continued to smoke regular cigarettes alongside electronic cigarettes. As appeared in Table 1.

### Factors Associated with the Knowledge, Attitude, and Practice of Electronic Cigarettes

The level of knowledge varied significantly across different age groups and marital statuses (p = 0.03) and (p = 0.013), respectively. A poor level of knowledge was most prevalent among participants aged 40-59 years (49.7%). Married smokers were more likely to have poor knowledge compared to their single, divorced, or widowed counterparts, with 85.4% of married participants showing poor knowledge and 73% of them showing good knowledge.

Furthermore, there was a statistically significant difference regarding the status of knowledge towards electronic cigarettes among participants of different educational backgrounds, where those with higher education (university degree) were more likely to have a good level of knowledge (72.6% of them with a good knowledge score vs 57.3%of them with poor knowledge) and those with lower education attainment were more likely to have poor level of knowledge (p=0.007). Similarly, the participants’ income was significantly associated with their level of knowledge. Those with a higher income (≥ 30.000 QR/ month) were more likely to have a good level of knowledge about electronic cigarettes (51.6% vs 40.4%) than those with a lower income (<10.000 QR/month, 16.1% vs 8.5%, p=0.033), as seen in Table 3. However, there was no statistically significant association between the participant’s background characteristics and Attitude towards electronic cigarettes.

**Table 3:** The Association Between the Participants’ Background Characteristics and Their Knowledge of Electronic Cigarettes in Qatar During 2020

Background Characteristic	Poor Knowledge (Score ˂ 3)	Good Knowledge (Score ≥ 3)	P- value
	n (%)	n (%)	
Age (years)			
18-23	4 (2.3)	7 (2.7)	0.030
24-39	81 (47.4)	156 (60.2)	
40-59	85 (49.7)	89 (34.4)	
≥60	1 (0.6)	7 (2.7)	
Sex			
Male	165 (96.5)	242 (94.2)	0.388
Female	6 (3.5)	15 (5.8)	
Nationality			
Qatari	18 (10.5)	40 (15.4)	0.139
Non-Qatari	153 (89.5)	219 (84.6)	
Marital status			
Single	23 (13.4)	62 (23.9)	0.013
Married	146 (85.4)	189 (73)	
Divorced	2 (1.2)	7 (2.7)	
Widowed	0 (0)	1 (0.4)	
Level of education			
Illiterate	3 (1.7)	1 (0.4)	0.007
Primary	7 (4.2)	3 (1.1)	
Preparatory	10 (5.8)	10 (3.9)	
Secondary	53 (31)	57 (22)	
University	98 (57.3)	188 (72.6)	
Employment			0.223
Employed	147 (86)	228 (88)	
Self-employed	19 (11.1)	16 (6.2)	
Unemployed	4 (2.3)	8 (3.1)	
Retired	1(0.6)	4 (1.5)	
Student	0 (0)	3 (1.2)	
Income			
< 10.000 QR	79 (51.6)	93 (40.4)	0.033
10.000 - < 30.000 QR	61 (39.9)	100 (43.5)	
≥ 30.000 QR	13 (8.5)	37 (16.1)	
Earlier quit attempt(s)			
Yes	161 (94.2)	244 (94.2)	0.204
No	10 (5.8)	15 (5.8)	
Daily tobacco smoker			
Yes	159 (93)	237 (91.5)	0.592
No	12 (7)	22 (8.5)	
Nicotine dependence			
Low	54 (35.5)	98 (37.8)	0.309
Low-moderate	52 (46.8)	59 (22.8)	
Moderate	56 (39.4)	86 (33.2)	
High	9 (36)	16 (6.2)	

**Abbreviation**: QR, Qatari Riyal.

The ever use of electronic cigarettes was most prevalent among participants with high education (university degree, 72.1%). In addition, the participants’ income was significantly associated with their ever use of electronic cigarettes. Those with income level (10.000 - < 30.000QR/month) were more likely to have used an electronic cigarette (44.7%), as appeared in Table 4.

**Table 4:** The Association Between the Participants’ Background Characteristics and Their Ever Use of Electronic Cigarettes in Qatar During 2020

Background Characteristic	No	Yes	P- value
	n (%)	n (%)	
Age (years)			
18-23	6 (2.6)	5 (2.3)	0.667
24-39	124 (53)	127 (58)	
40-59	100 (42.7)	82 (37.4)	
≥60	4 (1.7)	5 (2.3)	
Sex			
Male	225 (96.6)	204 (93.6)	0.141
Female	8 (3.4)	14 (6.4)	
Nationality			
Qatari	24 (10)	35 (16)	0.070
Non-Qatari	210 (90)	184 (84)	
Marital status			
Single	45 (19.2)	45 (20.5)	0.166
Married	187 (97.9)	166 (75.8)	
Divorced	2 (0.8)	7 (3.2)	
Widowed	0 (0)	1 (0.5)	
Level of education			
Illiterate	2 (0.8)	2 (1)	0.019
Primary	9 (3.8)	4 (1.8)	
Preparatory	13 (5.5)	7 (3.2)	
Secondary	75 (32)	48 (21.9)	
University	135 (57.7)	158 (72.1)	
Employment			
Employed	198 (84.6)	196 (89.5)	0.198
Self-employed	21 (9)	16 (7.3)	
Unemployed	7 (3)	5 (2.3)	
Retired	5 (2.1)	2 (0.9)	
Student	3 (1.3)	0 (0)	
Income			
< 10.000 QR	113 (54.6)	74 (37.8)	0.001
10.000 - < 30.000 QR	78 (37.7)	88 (44.9)	
≥ 30.000 QR	16 (7.7)	34 (17.3)	
Earlier quit attempt(s)			
Yes	219 (94.6)	121 (93.1)	0.673
No	15 (6.4)	9 (6.9)	
Daily tobacco smoker			
Yes	221 (94.4)	198 (90.4)	0.103
No	13 (5.6)	21 (9.6)	
Nicotine dependence			
Low	87 (37.2)	76 (34.7)	0.920
Low-moderate	62 (26.5)	57 (26)	
Moderate	73 (31.2)	73 (33.3)	
High	12 (5.2)	13 (6)	

### The Determinants of Good Knowledge and Use of Electronic Cigarettes

Concerning the participant’s background characteristics and a good knowledge of electronic cigarettes, having a high income (≥ 30.000 QR) was the only significant variable in the multiple logistic regression analyses, as seen in Table 5.

**Table 5:** Multiple Logistic Regression to Assess the Predictors of Good Knowledge on Electronic Cigarettes Among Adult Current Smokers at the Tobacco Control Center-Hamad Medical Corporation in Qatar During 2020 (N=453)

Explanatory Variable	Good Knowledge (Score ≥ 3)		
	Adjusted Odds Ratio [AOR]	95% CI of Exp (B)	P- value
Age (years)			
18-23	1.20	[0.22 −6.60]	0.823
24-39	1.45	[0.91–2.31]	0.110
40-59	1.00	-	-
≥60	4.05	[0.44–36.86]	0.211
Marital status			
Single	0.22	[0.02 −2.04]	0.181
Married	0.13	[0.01–1.13]	0.060
Others	1.00	-	-
Level of education			
Illiterate	1.00	-	-
Primary	1.01	[0.06–15.77]	0.990
Preparatory	2.73	[0.21–34.83]	0.434
Secondary	3.01	[0.28–31.61]	0.351
University	4.86	[0.47–50.09]	0.180
Income			
< 10.000 QR	1.00	-	-
10.000 - < 30.000 QR	1.19	[0.74–1.91]	0.460
≥ 30.000 QR	2.29	[1.08–4.84]	0.029

Concerning the participants’ background characteristics and having ever used an electronic cigarette, having a high income (≥ 30.000 QR) was the only significant variable detected in the multiple logistic regression analysis, as seen in Table 6.

**Table 6:** Multiple Logistic Regression to Assess the Predictors of Ever Use of Electronic Cigarettes Among Adult Current Smokers at the Tobacco Control Center-Hamad Medical Corporation in Qatar During 2020 (N=453)

Explanatory Variable	Ever Used Electronic Cigarette		
	Adjusted Odds Ratio [AOR]	95% CI of Exp (B)	P- value
Level of education			
Illiterate	1.00	-	-
Primary	0.40	[0.04–4.07]	0.440
Preparatory	0.47	[0.05–4.45]	0.511
Secondary	0.58	[0.07–4.34]	0.590
University	0.93	[0.12–6.83]	0.941
Income			
< 10.000 QR	1.00	-	-
10.000 - < 30.000 QR	1.49	[0.95–2.32]	0.070
≥ 30.000 QR	2.78	[1.41–5.48]	0.003

## Discussion

The novelty of this research lies in its comprehensive assessment of the knowledge, attitude, and practice regarding electronic cigarettes among current smokers attending smoking cessation clinics in Qatar. Unlike previous studies, this study provides a focused examination of a specific population in a unique context—smokers actively seeking to quit within a tobacco control centre in Qatar. Additionally, it explores the key factors associated with good knowledge and ever-use of electronic cigarettes. These findings offer new insights into how socio-demographic factors influence electronic cigarette awareness and behaviour in this population, contributing to the limited literature on electronic cigarette use in the region.

Remarkably, while nearly all individuals were aware of electronic cigarettes, a substantial proportion demonstrated limited knowledge, with 60.2% scoring ≥3 on the knowledge scale. Moreover, the study revealed prevalent negative sentiments toward electronic cigarettes, with a mean attitude score of −2.9. Intriguingly, despite these negative perceptions, almost half (48.3%) of the participants reported having tried electronic cigarettes, with 55.2% of them employing them as aids for smoking cessation. Importantly, higher income levels were associated with better knowledge about electronic cigarettes and a higher likelihood of having experimented with them.

The study’s findings indicate that a significant percentage of participants (60.2%) exhibited a good understanding of electronic cigarettes, contrasting with earlier research conducted among the Lebanese community, where only 36.7% of the participants demonstrated similar knowledge.^36^ Additionally, other studies among Malaysian adults^37^ and Pakistani university students^38^ revealed comparable levels of knowledge, with nearly half reporting good understanding. Furthermore, most participants in the current study reported having heard about electronic cigarettes, a trend mirrored in online surveys of Croatian adults^39^ and Pakistani university students,^38^ where high awareness levels were observed. Conversely, studies among adults in rural China^40^ and Jordanian adults^41^ showed lower levels of awareness.

The primary source of knowledge on electronic cigarettes for participants in the current study was identified as family members or friends, consistent with findings from a global online survey by Italian researchers,^42^ and the survey among university students in Pakistan.^38^ However, significant proportions of participants in the current study were unaware of certain aspects of electronic cigarettes, such as their nicotine content and the presence of carcinogens, which aligns with findings from other studies.^36^,^43–45^ Moreover, when asked about the composition of electronic cigarette vapour, a considerable portion of participants lacked knowledge, similar to findings from a national survey of American adults,^46^ and the online survey of Saudi adults,^43^ suggesting increased awareness and education on these topics across different populations.

Regarding attitude, our findings echo the results from the Lebanese community survey, where the majority (94.71%) shared similar sentiments.^36^ Regarding perceptions of addiction, a lower percentage (35%) of Lebanese adults believed electronic cigarettes were addictive.^36^ Similarly, Differing perspectives were noted among the Saudis,^47^ and Chinese university students^45^ regarding addiction. Additionally, a sizable portion of participants believed electronic cigarettes could aid in smoking cessation, aligning with perceptions observed among Lebanese pedestrians^36^ and the Saudi university students.^47^ However, Egyptian adults held a more doubtful perspective of electronic cigarettes’ effectiveness as quitting tools.^48^ Concerning Second-hand aerosol ‘s harm perception, varied opinions were noted among the Egyptians.,^49^ and Pakistani surveys,^38^ regarding the relative harm of electronic cigarettes compared to traditional cigarette.

While a significant portion of smokers in our study believed that electronic cigarettes enhance social acceptability, other surveys have found less consensus on this matter. For example, the Lebanese community-based survey showed fewer participants (43%) viewed electronic cigarettes as socially acceptable.^36^ Similarly, Saudi university students had differing opinions, with a substantial minority agreeing that electronic cigarette use boosts popularity among peers.^47^ Furthermore, attitudes towards the pleasure derived from using electronic cigarettes varied among our study participants, mirroring findings from the Lebanese survey.^47^ Regarding regulation and promotion, our study aligned with other studies in advocating for strict regulations on electronic cigarettes.^41^,^47^

In our investigation, around half of the smokers indicated previous use of electronic cigarettes, mirroring similar trends observed in a prior national survey in the United States.^46^ Similarly, a notable segment of Saudi university students employed electronic cigarettes as a cessation aid.^47^ However, the prevalence of electronic cigarette usage was lower among respondents in the surveys conducted in Lebanon,^36^ and Pakistan,^38^ with approximately one-tenth of smokers reporting electronic cigarette use. Conversely, only a minority of Chinese university students reported ever using electronic cigarettes.^45^ Among users, motivations for electronic cigarette usage varied among the studies, with a significant portion aiming to quit smoking altogether.^36^,^43^ Nonetheless, many users engaged in dual use, concurrently using both electronic cigarettes and traditional tobacco products.^41^,^47^ These findings underscore the multifaceted nature of electronic cigarette utilisation, highlighting both its potential as a tool for smoking cessation and the prevalence of simultaneous tobacco use among electronic cigarette users.

The study highlights the associations between age, education, income, and awareness levels, emphasising the role of socioeconomic status in accessing information and fostering awareness. Similar findings were observed in the Egyptian study.^49^ These results may be attributed to the increased curiosity of younger age groups toward new devices and their higher use of the Internet and social media, where electronic cigarettes are marketed. Moreover, highly educated individuals tend to be proficient with technology and have increased access to information sources due to their higher literacy levels.^49^ Additionally, having a higher financial status puts these participants in a social circle that possibly utilises electronic cigarettes, given their higher cost in comparison to regular cigarettes.

Furthermore, the study identified a poor level of electronic cigarette knowledge among married participants. In contrast, a similar study has determined that unmarried individuals suffer a lower level of health and higher mortality risk than their married peers.^50^ Moreover, a contradiction arises regarding the association between gender and electronic cigarette knowledge, as the current study did not find a significant gender disparity, in contrast to prior research that has suggested gender-based differences in awareness levels.^38^ This can be due to the limited number of female participants in the study in the context of the cultural biases that prohibit women from smoking or the social stigma that makes seeking smoking cessation more difficult.

Our study revealed that participants with higher education demonstrate a greater inclination towards using electronic cigarettes, echoing findings from similar research among Malaysian university students.^44^ However, contradictory results emerged from a survey of American adults, which failed to establish significant links between background characteristics and electronic cigarette use.^51^ In Qatar, where electronic cigarettes are prohibited, their purchase primarily occurs abroad due to legal constraints and higher costs compared to traditional tobacco products. The association between electronic cigarette use and higher income may stem from more significant financial resources for travel and access to online information among individuals with higher education levels, reflecting broader socioeconomic factors shaping consumption behaviors. Incorporating the findings from this study, the Ministry of Health and other stakeholders engaged in combating tobacco use can leverage several critical insights to inform policy and intervention strategies. First, the significant knowledge gaps revealed, especially concerning the health risks of electronic cigarettes, highlight the need for targeted educational campaigns that address these misconceptions among smokers. Given the high awareness but limited understanding of e-cigarette content and risks, the Ministry could develop public health initiatives that emphasise the potential harms of both direct use and second-hand vapour exposure. Moreover, the study’s identification of higher income and education levels as predictors of better e-cigarette knowledge suggests that interventions should be tailored to reach lower-income and less-educated smokers, who may be at a greater risk of using e-cigarettes without fully understanding the associated risks. Finally, given the substantial proportion of dual users (those using both traditional and electronic cigarettes), regulatory bodies might consider stricter regulations and controls on e-cigarette marketing and availability, particularly in Qatar, where their sale is prohibited, but usage persists. These steps could foster more comprehensive tobacco control strategies that align with national health priorities.Future research should explore the long-term effects of e-cigarettes and healthcare providers’ knowledge to improve cessation efforts. Stronger regulations and public awareness campaigns are needed to curb use, especially among vulnerable populations.

## Strengths

To our knowledge, this was the first national study in Qatar to examine e-cigarette knowledge, attitudes, and use. A key strength of the study was the use of random sampling, which allowed for a representative sample of the general population, minimizing selection bias. Additionally, achieving the calculated sample size ensured the study’s power and the reliability of its findings. The interview-based method of data collection further enhanced the completeness and accuracy of the information gathered. The use of a structured questionnaire, with established content and face validity, effectively assessed the three key domains: knowledge, attitudes, and practices. Moreover, conducting the study at the Tobacco Control Center, a government-subsidized referral center for smokers throughout Qatar, strengthened the external validity and generalizability of the results.

## Limitations

Despite its strengths, the study has certain limitations. The cross-sectional design does not allow for conclusions about causality between explanatory variables and e-cigarette knowledge, attitudes, and use. Additionally, the results provide a snapshot of the target population at the time of data collection, which may not capture the dynamic nature of these behaviors over time. Social desirability bias may have also affected the responses due to the use of an interview-based questionnaire in a healthcare setting, leading some participants to potentially misreport their true behaviors or attitudes. Finally, the relatively small number of current (n=7) and daily (n=1) e-cigarette users limited the ability to conduct further bivariate and multivariate analyses in this subgroup.

## Conclusion

Despite widespread awareness, significant gaps persist in understanding their contents and potential health implications. Negative attitudes towards electronic cigarettes prevail; however, a substantial portion of participants have experimented with them. Demographic factors such as age, marital status, educational background, and income level were found to be significantly associated with electronic cigarette knowledge and usage. These findings emphasise the need for targeted educational initiatives and regulatory interventions to address misconceptions and mitigate potential risks associated with electronic cigarette use among smokers in Qatar.

## References

[1] World Health Organisation. Tobacco. [cited May 25, 2023]. Available from: https://www.who.int/news-room/fact-sheets/detail/tobacco. Accessed August 26, 2025.

[2] Goodchild M, Nargis N, d’Espaignet ET. Global economic cost of smoking-attributable diseases. *Tobacco Control*. 2018;27(1):58–64. doi:10.1136/tobaccocontrol-2016-053305 28138063 PMC5801657

[3] AlMulla A, Mamtani R, Cheema S, et al. Epidemiology of tobacco use in Qatar: prevalence and its associated factors. *PLoS One*. 2021;16(4):e0250065. doi:10.1371/journal.pone.0250065 33857248 PMC8049255

[4] Al-Dahshan A, Al Muraikhi H, Musa S, et al. Prevalence and predictors of smoking cessation among smokers receiving smoking cessation intervention in primary care in Qatar: a 6-month follow-up study. *Front Public Health*. 2023;11:1166016. doi:10.3389/fpubh.2023.1166016 37275499 PMC10235512

[5] Sahu KK, Mishra AK, Lal A, Siddiqui AD, Abraham GM. From oncologist’s desk: hemato-oncological aspect of using vaporizers, E-cigarettes, and other electronic nicotine delivery systems (ENDS). *Ind J Hematol Blood Transfusion*. 2020;36:202–204. doi:10.1007/s12288-019-01177-8 PMC7042424 32158108

[6] Brown CJ, Cheng JM. Electronic cigarettes: product characterisation and design considerations. *Tobacco Control*. 2014;23(suppl 2):ii4–ii10. doi:10.1136/tobaccocontrol-2013-051476 24732162 PMC3995271

[7] Kesimer M. *Another Warning Sign: High Nicotine Content in Electronic Cigarettes Disrupts Mucociliary Clearance, the Essential Defense Mechanism of the Lung*. American Thoracic Society; 2019:1082–1084. 10.1164/rccm.201905-1080ED PMC6888647 31199664

[8] Kapan A, Stefanac S, Sandner I, Haider S, Grabovac I, Dorner TE. Use of electronic cigarettes in european populations: a narrative review. *Int J Environ Res Public Health*. 2020;17(6). doi:10.3390/ijerph17061971 PMC7142603 32192139

[9] O’Brien B, Knight-West O, Walker N, Parag V, Bullen C. E-cigarettes versus NRT for smoking reduction or cessation in people with mental illness: secondary analysis of data from the ASCEND trial. *Tobacco Induced Dis*. 2015;13(1):1–7. doi:10.1186/s12971-015-0030-2 PMC4374189 25814920

[10] Hartmann-Boyce J, McRobbie H, Butler AR, et al. Electronic cigarettes for smoking cessation. *Cochrane Database Systematic Rev*. 2021;2021(9):1. 10.1002/14651858.CD010216.pub6 PMC8438601 34519354

[11] McRobbie H, Bullen C, Hartmann‐Boyce J, Hajek P. Electronic cigarettes for smoking cessation and reduction. *Cochrane Database Systematic Rev*. 2014;2014(12):1. 10.1002/14651858.CD010216.pub2 25515689

[12] Malas M, van der Tempel J, Schwartz R, et al. Electronic cigarettes for smoking cessation: a systematic review. *Nicotine Tobacco Res*. 2016;18(10):1926–1936. doi:10.1093/ntr/ntw119 27113014

[13] Callahan-Lyon P. Electronic cigarettes: human health effects. *Tobacco Control*. 2014;23(suppl 2):ii36–ii40. doi:10.1136/tobaccocontrol-2013-051470 24732161 PMC3995250

[14] Fracol M, Dorfman R, Janes L, et al. The surgical impact of e-cigarettes: a case report and review of the current literature. *Arch Plastic Surg*. 2017;44(06):477–481. doi:10.5999/aps.2017.00087 PMC5801784 29069879

[15] Bayly JE, Bernat D, Porter L, O’Dare K, Choi K. Prevalence and characteristics of secondhand smoke and secondhand vapour exposure among youth. *Tobacco Control*. 2019;28(3):305–310. doi:10.1136/tobaccocontrol-2018-054265 30021870 PMC6338527

[16] Lachireddy K, Capon A. A systematic review of the health risks from passive exposure to electronic cigarette vapour. *Public Health Res Pract*. 2016;2016:1. 10.17061/phrp2621617 27734060

[17] Khouja JN, Suddell SF, Peters SE, Taylor AE, Munafò MR. Is e-cigarette use in non-smoking young adults associated with later smoking? A systematic review and meta-analysis. *Tobacco Control*. 2021;30(1):8–15. doi:10.1136/tobaccocontrol-2019-055433 PMC7803902 32156694

[18] Miech R, Patrick ME, Pm O, Johnston LD. E-cigarette use as a predictor of cigarette smoking: results from a 1-year follow-up of a national sample of 12th grade students. *Tobacco Control*. 2017;26(e2):e106–e11. doi:10.1136/tobaccocontrol-2016-053291 28167683 PMC5545162

[19] Berry KM, Fetterman JL, Benjamin EJ, et al. Association of electronic cigarette use with subsequent initiation of tobacco cigarettes in US youths. *JAMA Network Open*. 2019;2(2):e187794. doi:10.1001/jamanetworkopen.2018.7794 30707232 PMC6484602

[20] Vanyukov MM, Tarter RE, Kirillova GP, et al. Common liability to addiction and “gateway hypothesis”: theoretical, empirical and evolutionary perspective. *Drug Alcohol Dependence*. 2012;123:S3–S17. doi:10.1016/j.drugalcdep.2011.12.018 22261179 PMC3600369

[21] Vanyukov MM, Ridenour TA. Common liability to drug addictions: theory, research, practice. *Drug Alcohol Dependence*. 2012;123(0 1):S1. doi:10.1016/j.drugalcdep.2012.01.005 22310010 PMC3982606

[22] Watkins SL, Glantz SA, Chaffee BW. Association of noncigarette tobacco product use with future cigarette smoking among youth in the population assessment of tobacco and health (PATH) study, 2013-2015. *JAMA Pediatrics*. 2018;172(2):181–187. doi:10.1001/jamapediatrics.2017.4173 29297010 PMC5801043

[23] Silveira ML, Conway KP, Green VR, et al. Longitudinal associations between youth tobacco and substance use in waves 1 and 2 of the population assessment of tobacco and health (PATH) study. *Drug Alcohol Dependence*. 2018;191:25–36. doi:10.1016/j.drugalcdep.2018.06.018 30077053 PMC6239207

[24] Organization WH. Conference of the Parties to the WHO Framework Convention on Tobacco Control. Electronic nicotine delivery systems and electronic non-nicotine delivery systems (ENDS/ENNDS). Report by WHO. 2016. FCTC/COP/7/11, August 2016). Available from: https://www.who.int/fctc/cop. Accessed August 26, 2025.

[25] Rigotti NA. E-cigarette use and subsequent tobacco use by adolescents: new evidence about a potential risk of e-cigarettes. *JAMA*. 2015;314(7):673–674. doi:10.1001/jama.2015.8382 26284717

[26] Moph Q. https://assets.tobaccocontrollaws.org/uploads/legislation/Qatar/Qatar-TC-Law-2016.pdf. 2016 [cited Augest 10, 2023]. Available from: https://assets.tobaccocontrollaws.org/uploads/legislation/Qatar/Qatar-TC-Law-2016.pdf. Accessed August 26, 2025.

[27] Laws TC. Campaign for Tobacco-Free Kids. 2022 Available from: https://www.tobaccocontrollaws.org/legislation. Accessed August 26, 2025.

[28] Kurdi R, Al-Jayyousi GF, Yaseen M, Ali A, Mosleh N, Abdul Rahim HF. Prevalence, risk factors, harm perception, and attitudes toward e-cigarette use among university students in Qatar: a cross-sectional study. *Front Public Health*. 2021;9:682355. doi:10.3389/fpubh.2021.682355 34490180 PMC8417713

[29] corporation Hm. Tobacco control center [cited February 27, 2024]. Available from: https://hamad.qa/EN/Hospitals-and-services/QMI/TCC/Pages/default.aspx. Accessed August 26, 2025.

[30] Charan J, Biswas T. How to calculate sample size for different study designs in medical research? *Ind J Psychol Med*. 2013;35(2):121–126. doi:10.4103/0253-7176.116232 PMC3775042 24049221

[31] Palipudi KM, Group GC, Mbulo L, et al. Awareness and current use of electronic cigarettes in Indonesia, Malaysia, Qatar, and Greece: findings from 2011–2013 global adult tobacco surveys. *Nicotine Tobacco Res*. 2015;18(4):501–507. doi:10.1093/ntr/ntv081 PMC5100820 25895951

[32] Al-Shami AM, Elsayed TM, Elkalmi RM, et al. Knowledge, attitude and practice of smoking among pharmacy students: findings from a public university. *J Clin Diagnostic Res*. 2018;12(12):1.

[33] Zhu S-H, Gamst A, Lee M, Cummins S, Yin L, Zoref L. The use and perception of electronic cigarettes and snus among the US population. *PLoS One*. 2013;8(10):e79332. doi:10.1371/journal.pone.0079332 24250756 PMC3824062

[34] Filippidis FT, Laverty AA, Gerovasili V, Vardavas CI. Two-year trends and predictors of e-cigarette use in 27 European Union member states. *Tobacco Control*. 2017;26(1):98–104. doi:10.1136/tobaccocontrol-2015-052771 27220621 PMC5256312

[35] The Brief Tobacco Intervention Training Programe. Fagerstorm Test for Nicotine Dependance [cited May 29, 2024]. Available from: https://www.aarc.org/wp-content/uploads/2014/08/Fagerstrom_test.pdf. Accessed August 26, 2025.

[36] Aghar H, El-Khoury N, Reda M, et al. Knowledge and attitudes towards E-cigarette use in Lebanon and their associated factors. *BMC Public Health*. 2020;20:1–18. doi:10.1186/s12889-020-8381-x 32111186 PMC7049178

[37] Hafiz A, Rahman MM, Jantan Z. *Factors Associated with Knowledge, Attitude and Practice of e-Cigarette Among Adult Population in KOSPEN Areas of Kuching District, Sarawak, Malaysia*. 2019.

[38] Sarfraz M, Khan HAR, Urooba A, et al. Awareness, use and perceptions about E-cigarettes among adult smokers in Karachi, Pakistan. *JPMA J Pakistan Med Assoc*. 2018;68(1):147. 29371741

[39] Puharić Z, Smola V, Žulec M, Grabovac S, Puharić F, Petričević N. Knowledge, Attitudes and Use of E-Cigarettes. *Arch Psych Res*. 2021;57(1):5–14. doi:10.20471/may.2021.57.01.01

[40] Wang F, He Y, Zhang R, Zeng Q, Zhao X. Combination therapy of metformin plus dipeptidyl peptidase-4 inhibitor versus metformin plus sulfonylurea and their association with a decreased risk of cardiovascular disease in type 2 diabetes mellitus patients. *Medicine*. 2017;96(36):e7638. doi:10.1097/MD.0000000000007638 28885325 PMC6393015

[41] Abdel-Qader DH, Al Meslamani AZ. Knowledge and beliefs of Jordanian community toward E-cigarettes: a national survey. *J Community Health*. 2021;46:577–586. doi:10.1007/s10900-020-00896-8 32772206

[42] Gupta V, Sharma M, Srikant N, Manaktala N. Assessment of knowledge of use of electronic cigarette and its harmful effects among young adults. *Open Med*. 2020;15(1):796–804. doi:10.1515/med-2020-0224 PMC7711978 33336037

[43] Alfaraj DN, Alessa YZ, Abdulatif FAA, Alshorafa JM, Alshakhs MA, Butayan HAA. Knowledge and perception of risks and use of e-cigarettes (vaping) among adults in the eastern province of Saudi Arabia. *Int J Med Res Health Sci*. 2019;8(12):17–31.

[44] Jaafar H, Razi NAM, Mohd TAMT, et al. Knowledge, attitude and practice on electronic cigarette and their associated factors among undergraduate students in a public university. *IIUM Med J Malaysia*. 2021;20(2). doi:10.31436/imjm.v20i2.506

[45] Wang W, Lu M, Cai Y, Feng N. Awareness and use of e-cigarettes among university students in Shanghai, China. *Tobacco Induced Dis*. 2020;18:18. doi:10.18332/tid/118722 PMC7516251 32994762

[46] Tan AS, Mello S, Sanders‐Jackson A, Bigman CA. Knowledge about chemicals in e‐cigarette secondhand vapor and perceived harms of exposure among a national sample of US adults. *Risk Anal*. 2017;37(6):1170–1180. doi:10.1111/risa.12676 27595498 PMC5567734

[47] Aqeeli AA, Makeen AM, Al Bahhawi T, et al. Awareness, knowledge and perception of electronic cigarettes among undergraduate students in Jazan Region, Saudi Arabia. *Health Soc Care Community*. 2022;30(2):706–713. doi:10.1111/hsc.13184 32974976

[48] Dwedar I, Ruby D, Mostafa A. A survey exploring knowledge and beliefs about electronic cigarettes between health care providers and the general population in Egypt. *Int J Chronic Obstructive Pulmonary Dis*. 2019;1943–1950. doi:10.2147/COPD.S214389 PMC6719839 32021137

[49] Abo-Elkheir OI, Sobh E. Knowledge about electronic cigarettes and its perception: a community survey, Egypt. *Resp Res*. 2016;17:1–7. doi:10.1186/s12931-016-0365-0 PMC4869382 27183972

[50] Robards J, Evandrou M, Falkingham J, Vlachantoni A. Marital status, health and mortality. *Maturitas*. 2012;73(4):295–299. doi:10.1016/j.maturitas.2012.08.007 23007006 PMC3635122

[51] Regan AK, Promoff G, Dube SR, Arrazola R. Electronic nicotine delivery systems: adult use and awareness of the ‘e-cigarette’in the USA. *Tobacco Control*. 2013;22(1):19–23. doi:10.1136/tobaccocontrol-2011-050044 22034071

